# High diversity of *Rickettsia* spp., *Anaplasma* spp., and *Ehrlichia* spp. in ticks from Yunnan Province, Southwest China

**DOI:** 10.3389/fmicb.2022.1008110

**Published:** 2022-10-13

**Authors:** Miao Lu, Junhua Tian, Wen Wang, Hongqing Zhao, Hai Jiang, Jizhou Han, Wenping Guo, Kun Li

**Affiliations:** ^1^Chinese Center for Disease Control and Prevention, National Institute for Communicable Disease Control and Prevention, Beijing, China; ^2^Wuhan Center for Disease Control and Prevention, Wuhan, Hubei, China; ^3^Dehong Center for Disease Control and Prevention, Dehong Dai and Jingpo Autonomous Prefecture, Yunnan, China; ^4^Department of Pathogenic Biology, College of Basic Medicine, Chengde Medical University, Chengde, Hebei, China; ^5^Tianjin Key Laboratory of Food and Biotechnology, Tianjin University of Commerce, Tianjin, China

**Keywords:** *Rickettsia*, anaplasma, ehrlichia, Yunnan Province, *Candidatus Rickettsia* shennongii

## Abstract

*Rickettsia*, *Anaplasma*, and *Ehrlichia* belonging to the order Rickettsiales are causative agents of tick-borne diseases in humans. During 2021, 434 ticks including *Rhipicephalus microplus* and *R. haemaphysaloides* were collected from three sampling sites in Yunnan Province, Southwest China, and analyzed for the presence of these bacteria. Nine bacterial species were identified, including two *Rickettsia* spp., three *Anaplasma* spp., and four *Ehrlichia* spp., some of which are potential human pathogens. Genetic and phylogenetic analysis on 16S rRNA, *gltA*, *groEL*, *ompA*, *ompB*, and *sca4* genes indicated the presence of a novel spotted fever group *Rickettsia* (SFGR) named “*Candidatus Rickettsia* shennongii” in six of the 38 *R. haemaphysaloides* ticks from two locations, Dehong Autonomous Prefecture and Honghe City. Another SFGR species, *Candidatus Rickettsia* jingxinensis was detected in ticks from all three sites, with an overall positive rate of 62.67%. Three other human pathogenic species, *Anaplasma ovis* (1.38%, 6/434)*, Ehrlichia canis* (16.36%, 71/434), and *E. chaffeensis* (0.23%, 1/434) were detected in these ticks and characterized. Moreover, *Ehrlichia* sp. (4.84%, 21/434), *E. minasensis* (7.37%, 32/434), *A. marginale* (6.91%, 30/434), and *Cadidatus* Anaplasma boleense (1.15%, 5/434) were detected in *R. microplus* ticks, for which pathogenicity to humans remains to be determined. The results reveal the remarkable diversity of Rickettsiales bacteria in ticks from Yunnan Province, Southwest China. The high infection rate of some human pathogenic bacteria in ticks may indicate potential infection risk in humans, and it highlights the need for surveillance in local populations.

## Introduction

*Anaplasma* spp. and *Ehrlichia* spp. belonging to order Rickettsiales are tick-borne intracellular bacteria, many of which are important pathogens of livestock. *Anaplasma* spp., *Ehrlichia* spp., as well as *Rickettsia* spp. bacteria also occasionally infect humans. Therefore, these bacteria are important for both veterinary and human public health (Gondard et al., 2017). SFGR represents a large group within the genus *Rickettsia* including *Rickettsia rickettsii*, *R. conorii*, *R. australis*, *R. honei*, *R. japonica, R. africae*, *R. sibirica*, etc. (Stenos et al., 2005). They are widely distributed and many of them are etiological agents of known human diseases, such as Rocky Mountain spotted fever (RMSF), Astrakhan fever, Mediterranean spotted fever, Indian tick typhus, Queensland tick typhus, Flinders Island spotted fever, Japanese spotted fever, African tick bite fever, and North Asian tick-typhus, etc. (Sentausa et al., 2012; Graham et al., 2017; Rudakov et al., 2019; Zazueta et al., 2021). Of those, *R. rickettsii* mainly distributes in North and South America and was reported to infect more than 4,000 patients during 2009–2019 at Mexico-United States Border (Zazueta et al., 2021). From 1997 to 2004, 415 children cases of Mediterranean spotted fever caused by *R. conorii* were recorded in Sicily, Italy (Colomba et al., 2006). Meanwhile, only sporadic infection cases were reported for *R. australis*, *R. honei* (in Australia), *R. africae* (in Africa), *R. japonica* (Asia), and other SFG members (Parola et al., 2013). For the genus *Anaplasma*, *Anaplasma marginale*, *A. centrale*, and *A. bovis* are common pathogens causing severe or mild anaplasmosis in ruminants, especially cattle (Rjeibi et al., 2018). Meanwhile, *A. phagocytophilum*, *A. bovis*, and *A. capra* are confirmed human pathogens (Li H. et al., 2015; Ismail and McBride, 2017; Lu et al., 2019). In the past decades, a total of 110 infection cases of *A. phagocytophilum* were reported (Dumic et al., 2022). Among *Ehrlichia* species, *E. ruminantium* and *E. minasensis* are known to infect cattle, causing severe fever, anemia, and thrombocytopenia (Peter et al., 2020). *Ehrlichia chaffeensis*, *E. ewingii*, *E. muris*, *E. muris.* Subsp. *eauclairensis*, and *E. canis* are reported to infect humans, with syndromes ranging from febrile to multiple organ failure (Saito and Walker, 2016; Pritt et al., 2017). From 2012–2016, 6,786 cases of *E. chaffeensis* infection were reported in the United States (Mogg et al., 2020).

Located in southwest China, Yunnan Province covers a vast area with diverse climates and unique biodiversity resources. The extraordinary biological and ecological diversity of this area makes the extensive diversity of Rickettsiales bacteria possible. In recent decades, much attention has been paid to Rickettsiales bacteria circulating in this area. Numerous studies have been performed and many bacterial species belonging to the order Rickettsiales have been characterized in Yunnan Province (Liang et al., 2012; Liu et al., 2020; Jiao et al., 2021). One study revealed high positive rates of SFGR in domestic animals (goats, dogs, and cattle), and proved the existence of *R. heilongjiangensis* and a distinct *Rickettsia* (Liang et al., 2012). Another study reported the presence of *R. raoultii* and *Ca.* R. jingxinensis in ticks from Yunnan Province (Liu et al., 2020). In Jiao et al. (2021) detected multiple tick-borne pathogens circulating in *Rhipicephalus microplus* ticks from Yunnan, including *Ca.* R. jingxinensis, *A. marginale*, and *Coxiella burnetii*. Furthermore, *A. capra*, *A. phagocytophilum*, and *Candidatus* Neoehrlichia mikurensis were also detected in *R. microplus* ticks from Yunnan Province (Jiao et al., 2021). Although many studies on tick-borne Rickettsiales bacteria have been performed in Yunnan Province, further exploration is still needed. To improve our knowledge on the biodiversity and epidemiology of Rickettsiales bacteria, we collected ticks from goats and cattle in three locations of Yunnan Province, and characterized *Rickettsia*, *Anaplasma*, and *Ehrlichia* in them.

## Materials and methods

### Sample collection and processing

**Figure 1:**
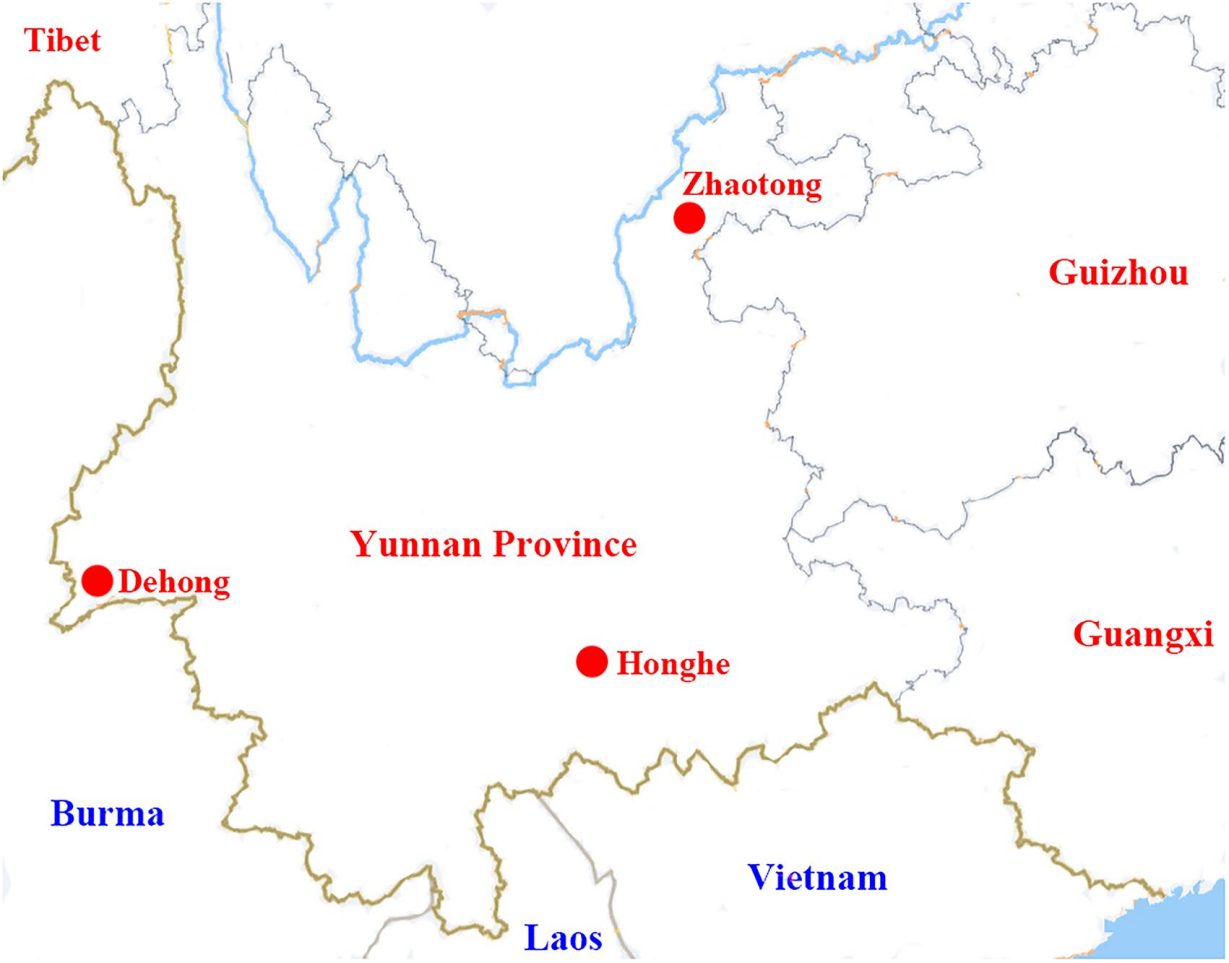
A map showing the locations of Dehong, Honghe, and Zhaotong in Yunnan Province, where the tick samples were collected.

During 2021, ticks were collected from three locations in Yunnan Province: Ruili county-level city of Dehong Dai-Jingpo Autonomous Prefecture (97.85°E, 24.01°N), Zhaoyang District of Zhaotong city (103.71°E, 24.32°N), and Shiping county of Honghe city (102.49°O, 23.71°N; Figure 1). Ticks were collected from cattle and goats, then brought to China CDC alive. Tick species were morphologically characterized by a sophisticated arthropod taxonomist based on the characteristics of the capitula, body, legs, anal groove, and caudal appendage (Namgyal et al., 2021). For further confirmation, randomly selected ticks were undergone molecular analysis by sequencing the mitochondrial cytochrome oxidase I (*COI*) gene sequences (Lu et al., 2013).

### DNA extraction, PCR detection, and amplification of key genes

After washing three times with sterile phosphate-buffered saline (PBS), ticks were individually homogenized in 500 μl of PBS using a Mixer Mill MM 400 (Retsch, Hann, Germany). The total DNA of each tick was extracted in 80 μl of eluate using a Mollusk DNA Extraction Kit (Omega Bio-Tek, United States) according to the manufacturer’s instructions.

Each tick DNA sample was screened by PCR for the presence of bacterial DNA including *Rickettsia* spp., *Anaplasma* spp., and *Ehrlichia* spp. Rickettsial DNA was detected by nested PCR as described previously using primers targeting the outer membrane protein A (*ompA*) gene, resulting in amplification of a 755-bp fragment (Lu et al., 2017). The DNA samples were also screened for *Anaplasma* and *Ehrlichia* DNA as described previously, amplifying a 550–850 bp fragment of the 16S rRNA gene (Guo et al., 2016). Negative control with distilled water in the PCR master mixture and positive control (DNA of *R. felis*, *A. marginale* and *E. canis*, respectively) were included in each test. After 1% agarose gel electrophoresis, amplification products were subjected to sequencing.

For further characterization and phylogenetic analysis of the detected bacterial strains, the partial sequences of the *gltA* gene (citrate synthase), the *groEL* gene (heat shock protein), and a long fragment of the 16S rRNA gene were obtained for representative *Rickettsia*, *Anaplasma*, and *Ehrlichia* positive samples. Additionally, nearly complete sequences of *ompB* and *sca4* genes were obtained for the putative novel *Rickettsia* species. The primers used were described previously (Roux and Raoult, 2000; Sekeyova et al., 2001; Kang et al., 2014; Guo et al., 2016; Lu et al., 2017) and they are listed in Supplementary Table S1. All recovered sequences have been deposited in the GenBank database (GenBank numbers listed in Supplementary Table S2).

### Genetic and phylogenetic analysis of obtained sequences

Sequences obtained in this study were analyzed by BLASTn^1^ and Clustal W within Molecular Evolutionary Genetics Analysis (MEGA) software, version 7.0 (Kumar et al., 2016). Phylogenetic analysis was also performed using PhyML 3.0. Confidence values for each branch of the phylogenetic trees were determined by bootstrap analysis with 1,000 replicates. Sequences recovered in this study were aligned with the reference sequences retrieved from the GenBank database.

## Results

### Sample collection

During March to July 2021, 434 ticks (397 *R. microplus* and 37 *R. haemaphysaloides*) were collected from three locations in Yunnan Province. The three sampling locations (Ruili County-level City of Dehong Dai-Jingpo Autonomous Prefecture, Zhaoyang District of Zhaotong City, and Shiping County of Honghe City) were shown in Figure 1. Tick species were initially determined by morphological examination and then further determined by amplification and analysis of the *COI* gene (Lu et al., 2013). Except for four nymphs from Zhaotong, all ticks were adult, and most were fully or partially engorged. The *COI* sequences of ticks have been submitted to GenBank. The Accession numbers are OM959242-OM959313, OM959315-OM959321, and OM977035-OM977043. The tick species, quantity, and vertebrate hosts in different sampling sites were shown in Supplementary Table S3.

### *Rickettsia* spp.

Nested PCR targeting the conserved domain of the *ompA* gene was performed to screen for *Rickettsia* in the collected tick samples. Based on agarose gel electrophoresis, DNA sequencing, and comparison with BLASTn, two *Rickettsia* species were initially identified: *Ca.* R. jingxinensis and a putative novel species. For further characterization, the 16S rRNA gene (1,172 bp), as well as the *gltA* (1,004 bp) and *groEL* (1038–1,042 bp) genes were successfully amplified from the total tick DNA samples.

DNA of *Ca.* R. jingxinensis was detected in ticks from all three sites, with strikingly high positive rates varying from 37.50 to 84.69% (48 of 128 ticks from Dehong, 166 of 196 ticks from Zhaotong, and 70 of 110 ticks from Honghe; Table 1). All *Ca.* R. jingxinensis strains were detected in *R. microplus* ticks except for one from *R. haemaphysaloides.* All the *ompA* sequences shared 100% identity with *Ca.* R. jingxinensis isolate Meixian-Hl-107 (MH932061.1) and Xian-Hl-79 (MH932069.1) reported in *Haemaphysalis longicornis* from ShaanXi Province, Northern China (Guo et al., 2019). The 16S rRNA, *gltA* and *groEL* genes shared 99.90–100% identity with each other in reference to homologous genes. The 16S rRNA and *gltA* gene sequences were 100 and 99.90% identical to other *Ca.* R. jingxinensis strains from China. Meanwhile, the *groEL* sequences shared highest 99.90% identity with Uncultured *Rickettsia* sp. clone tick28 (ON409665) and Uncultured *Rickettsia* sp. clone tick26 (ON409664) we previously identified in Ngawa, Sichuan Province, which actually also represent *Ca.* R. jingxinensis strains.

**Table 1:** Prevalence of Rickettsiales bacteria in ticks in Dehong, Zhaotong, and Honghe cities of Yunnan Province, Southwest China.

	Dehong		Zhaotong	Honghe		Total (%)
	***R. microplus*** (%)	***R. haemaphysaloides*** (%)	***R. microplus*** (%)	***R. microplus*** (%)	***R. haemaphysaloides*** (%)	
*Ca. Rickettsia* jingxinensis	48/97 (49.48)^b^	0/31 (0.00)	166/196 (84.69)^c^	69/103 (66.99)^d^	1/7 (14.29)	284/434 (65.44)
*Ca. Rickettsia* shennongii	0/97 (0.00)	3/31 (9.68)	0/196 (0.00)	0/103 (0.00)	3/7 (42.86)	6/434 (1.38)
*Ca.* Anaplasma boleense	2/97 (2.06)	0/31 (0.00)	1/196 (0.51)	2/103 (1.94)	0/7 (0.00)	5/434 (1.15)
*Anaplasma ovis*	1/97 (1.03)	1/31 (3.22)	0/196 (0.00)	0/103 (0.00)	4/7 (57.14)	6/434 (1.38)
*Anaplasma marginale*	6/97 (6.19)	0/31 (0.00)	8/196 (4.08)	16/103 (15.53)	0/7 (0.00)	30/434 (6.91)
*Ehrlichia canis*	0/97 (0.00)	0/31 (0.00)	71/196 (36.22)	0/103 (0.00)	0/7 (0.00)	71/434 (16.36)
*Ehrlichia chaffeensis*	0/97 (0.00)	0/31 (0.00)	1/196 (0.51)	0/103 (0.00)	0/7 (0.00)	1/434 (0.23)
*Ehrlichia minasensis*	0/97 (0.00)	0/31 (0.00)	0/196 (0.00)	32/103 (31.07)	0/7 (0.00)	32/434 (7.37)
*Ehrlichia* sp.	15/97 (15.46)	0/31 (0.00)	0/196 (0.00)	14/103 (13.59)	0/7 (0.00)	21/434 (4.84)

a Positive samples/total samples.

b Five were co-infected with *A. marginale* and four were co-infected with *Ehrlichia* sp.

c Fifty-four samples were co-infected with *E. canis*.

d Nine were co-infected with *E. minasensis*, two were co-infected with *Ehrlichia* sp., and nine were co-infected with both *E. minasensis* and *Ehrlichia* sp.

**Figure 2:**
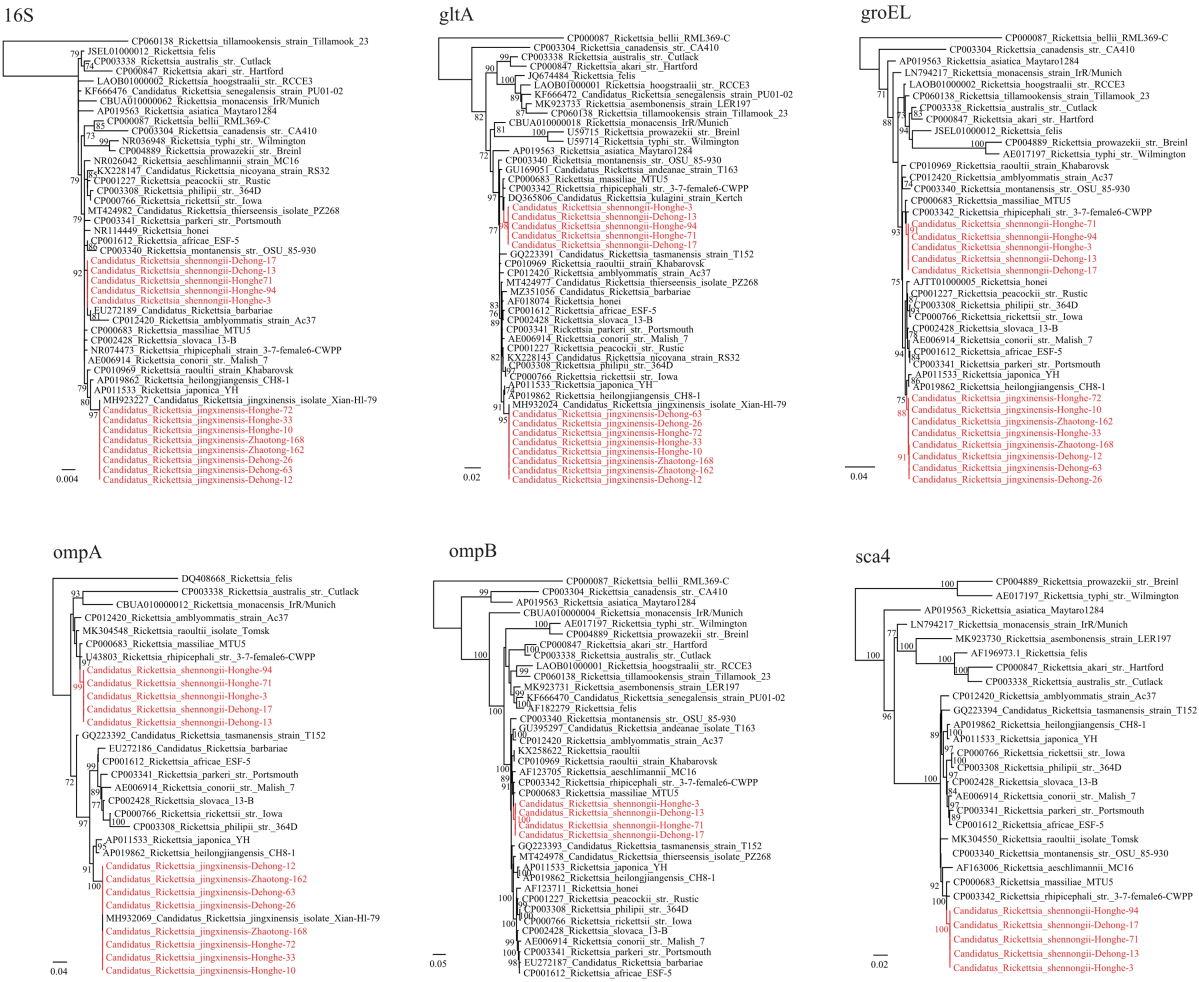
Phylogenetic trees constructed by the PhyML 3.0 software based on the nucleotide sequences of 16S rRNA (1,172 bp), *groEL* (1,038–1,042 bp), *gltA* (1,004 bp), *ompA* (664–712 bp), *ompB* (4,704–4,707 bp) and *sca4* (2,560–2,566 bp) genes of *Rickettsia* strains. The bootstrap values were shown on the nodes. Red: the strains identified in this study.

Notably, a putative novel SFGR species was identified in *R. haemaphysaloides* ticks from Dehong (3/31, 9.68%) and Honghe (3/7, 42.86%; Table 1). The *ompA* sequences of these were 100% identical to each other, and they shared 98.31% identity with *R. rhipicephali* str. 3-7-female6-CWPP (CP003342.1) and 98.17% with *R. massiliae* MTU5 (CP000683.1). The 16S rRNA (1,172 bp) gene sequences of all strains from Dehong and Honghe were identical, despite the geographic separation, and they shared 100.0% identity with *Rickettsia* sp. strain HB-9543 N2 (MT434770), 99.91% identity with *R. raoultii* isolate Tomsk (MK304546) and *R. conorii* str. Malish 7 (AE006914.1). The *gltA* (1,004 bp) gene sequences were found to be 99.70–99.80% identical to *Rickettsia* sp. strain HB-9543 N2 (MT434984), 99.30–99.40% to *R. massiliae* MTU5 (CP000683.1) and *R. rhipicephali* str. HJ#5 (CP013133). Regarding the *groEL* (1,042 bp) gene, all sequences shared high similarity with *R. rhipicephali* str. HJ#5 (CP013133, 99.42%) and *R. rhipicephali* str. 3-7-female6-CWPP (CP003342; 99.33%). For further confirmation, the *ompB* (4704–4,707 bp) and *sca4* (2560–2,566 bp) genes were successfully recovered. Similar to other genes, the *ompB* gene shared 99.68–99.77% similarity to *Rickettsia* sp. strain HB-9543 N2 (MT434988) and 98.14–98.22% similarity with *R. rhipicephali* str. 3-7-female6-CWPP (CP003342.1), while the *sca4* gene shared 99.73–99.96% identity with *Rickettsia* sp. strain HB-9543 N2 (MT434990) and 98.10–98.37% identity to *R. rhipicephali* str. 3-7-female6-CWPP (CP003342). Notably, the *sca4* gene of all strains from Honghe City has an additional six-nucleotide insertion (AAGAAA). In the phylogenetic trees, all six genes of this *Rickettsia* formed a distinct clade closely related to *R. rhipicephali and R. massiliae* (Figure 2).

According to the gene sequence-based criteria for the identification of a new *Rickettsia* species, a novel species should exhibit at most one of the following degrees of sequence similarity when comparing to validated *Rickettsia* species: ≥99.8 for the 16S, ≥99.9% for the *gltA* genes, and, when available, ≥98.8, ≥99.2, and ≥ 99.3% for the *ompA* and *ompB* and *sca4* genes, respectively (Fournier et al., 2003). As indicated above, BLASTn shows that the 16S, *gltA*, *ompA*, *ompB*, and *sca4* sequences of this *Rickettsia* have highest 99.91%, 99.30–99.40, 99.42, 98.31%, 98.14–98.22%, and 98.10–98.37% similarity to validated *Rickettsia* species, respectively (Supplementary Table S4). All the sequences match this criterion except the 16S gene has a similarity higher than 99.8%. This result clearly supports that they represent a novel *Rickettsia* species. Herein we name it “*Candidatus Rickettsia* shennongii” to memorize Shennong, a great master of agronomy and herbology in Chinese myth.

### *Anaplasma* spp.

**Figure 3:**
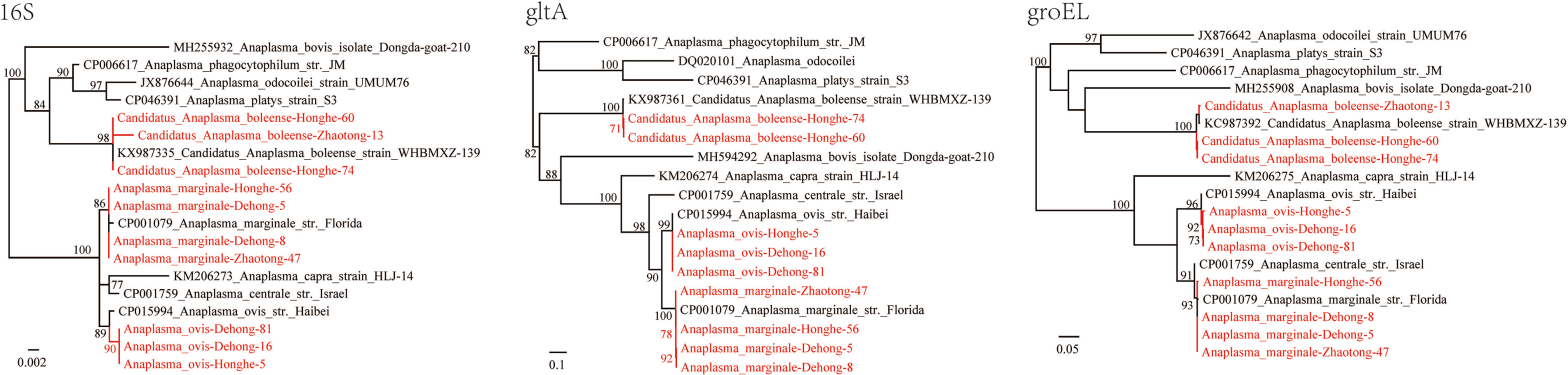
Phylogenetic trees constructed by the PhyML 3.0 software based on the nucleotide sequences of 16S rRNA (854–1,206 bp), *gltA* (539 bp), and *groEL* (846 bp) genes of *Anaplasma* strains. The bootstrap values were shown on the nodes. Red: the strains identified in this study.

Three *Anaplasma* species were identified; *Candidatus* Anaplasma boleense, *A. ovis*, and *A. marginale*. DNA of *Ca.* A. boleense was detected in *R. microplus* ticks from Dehong, Zhaotong, and Honghe, with positive rates of 2.06% (2/97), 0.51% (1/196), and 1.94% (2/103), respectively (Table 1). The 16S rRNA (855 bp), *gltA* (672 bp), and *groEL* (857 bp) genes share 99.65–100, 99.11%, and 98.95–100% similarity with *Ca.* A. boleense strains reported in other locations of China. *Anaplasma ovis* was detected in ticks from Dehong and Honghe, mostly from *R. haemaphysaloides*. All the three genes (16S rRNA, 854–1,206 bp; *gltA*, 539 bp; *groEL*, 846 bp) are 100% identical to previously reported *A. ovis* sequences, and they are closely clustered with other *A. ovis* strains in the phylogenetic trees (Figure 3). As a widely distributed animal pathogen, *A. marginale* was detected in *R. microplus* ticks from all three locations, with prevalence rates from 4.08 to 15.53% (Table 1). All three genes (16S rRNA: 854–1,206 bp, *gltA*: 857 bp, *groEL*: 926 bp) share 100% identity with previously reported *A. marginale* sequences. To be noticed, it has been observed that the detection rates of *Anaplasma* spp. in ticks from the environment and those removed from livestock are different (von Fricken et al., 2021). Given all ticks in this study were removed from cattle and goats, the detection rates might be influenced.

### *Ehrlichia* spp.

Of the 434 ticks screened from three locations, 125 (28.80%) were positive for *Ehrlichia* including four species: *E. minasensis*, *E. canis*, *E. chaffeensis*, and *Ehrlichia* sp. (Table 1). In *R. microplus* ticks from both Dehong and Honghe city, an *Ehrlichia* sp. species closely related to *Ehrlichia* sp. strain WHBMXZ-43 was identified, which was first identified in *R. microplus* ticks from Wuhan city, South-Central China. The 16S rRNA genes of the detected strains share 99.84–99.91% identity with this *Ehrlichia*, while both *gltA* and *groEL* genes share 100% identity with this strain.

**Figure 4:**
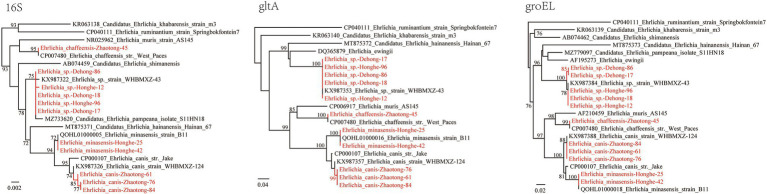
Phylogenetic trees constructed by the PhyML 3.0 software based on the nucleotide sequences of 16S rRNA (1,198–1,406 bp), *gltA* (864–973 bp), and *groEL* (1,115 bp) genes of *Ehrlichia* strains. The bootstrap values were shown on the nodes. Red: the strains identified in this study.

In *R. microplus* ticks from Honghe city, *E. minasensis* was detected, with a positive rate of 31.07% (32/103). The 16S rRNA, *gltA*, and *groEL* gene sequences all share 100% identity with *E. minasensis* strain B1 isolated from cattle in Brazil. In the phylogenetic trees based on these three genes, the two *E. minasensis* strains (Honghe-25 and Honghe-42) were both located in the same clade with *E. minasensis* strain B11 (Figure 4). This result indicated the prevalence and the high genetic conservation of *E. minasensis* in Yunnan Province.

Of the 196 *R. microplus* ticks tested from Zhaotong city, two *Ehrlichia* species were detected: *E. canis* (71/196, 36.22%) and *E. chaffeensis* (1/196, 0.51%; Table 1). The prevalence in male, female, and nymph ticks was shown in Supplementary Table S5. The 16S rRNA gene sequences of detected *E. canis* strains (1,406 bp) share 99.57% similarity with *E. canis* clone CuD125 (MK507008.1) and *E. canis* strain YZ-1 (CP025749.1), etc. The *gltA* and *groEL* sequences are 100% identical between the detected strains. The *gltA* (973 bp) sequences are 97.84% identical to *E. canis* strain YZ-1 (CP025749.1) and *E. canis* isolate T85D7 (MW382940.1), while the *groEL* sequences share 98.66% identity with *E. canis* strain Mossesane (MG953295.1). Phylogenetic analysis clearly revealed that all strains are assembled with other *E. canis* strains (Figure 4). Additionally, an *E. chaffeensis* strain was detected in a single *R. microplus* tick. All 16S rRNA, *gltA*, and *groEL* genes share 100% similarity with *E. chaffeensis* str. West Paces, *E. chaffeensis* Arkansas, and other previously reported *E. chaffeensis* strains. These appear to be the only *gltA* and *groEL* sequences of *E. chaffeensis* from China submitted to GenBank.

## Discussion

Yunnan Province is a widely recognized biodiversity hotspot in China and worldwide (Li R. et al., 2015). In the present study, we revealed the extensive diversity and high positive rate of Rickettsiales bacteria in ticks from Yunnan Province. Nine bacterial species belonging to the genus *Rickettsia*, *Anaplasma*, and *Ehrlichia* were detected, including a novel SFGR species. Our result may contribute to the current knowledge of the biodiversity of Rickettsiales bacteria circulating in this area.

*Rickettsia* spp. have been recognized as pathogens of potential public health importance. Herein, the DNA of *Ca.* R. jingxinensis was detected in all three locations with extremely high positive rates varying from 37.50 to 84.69%. *Cadidatus Rickettsia* jingxinensis is a SFGR first identified in *Ha. longicornis* from northeast China in 2016 (Liu et al., 2016). It has since been detected in ticks from multiple provinces in China, including Shaanxi, Guangxi, Sichuan, and Yunnan (Guo et al., 2019; Liu et al., 2020; Jiao et al., 2021; Lu et al., 2022). Furthermore, it was also reported in Korea, Thailand, and India, spanning from East Asia to South Asia (Takhampunya et al., 2019; Bang et al., 2021) (GenBank No. MN463681-MN463688). Previous studies revealed strikingly high positive rates of this *Rickettsia* in certain areas (for instance, 44.4–69.7% in *H. longicornis* from Shaanxi and 24.61%in *R. microplus* from Yunnan; Guo et al., 2019; Jiao et al., 2021). Our findings confirmed the widespread circulation of *Ca.* R. jingxinensis in China, as well as its high positive rate in ticks removed from livestock. Because most ticks were fully or partially engorged in the present work, it is not clear whether the *Rickettsia* DNA was from the blood meal or the tick itself. In another word, the possibility remains that the livestock in these locations may be infected by *Ca.* R. jingxinensis. These results remind us that the potential risk of *Ca.* R. jingxinensis infecting animals should be considered, and more attention should be paid to its role in human/animal diseases.

In *R. haemaphysaloides* ticks from Dehong and Honghe, a novel SFGR species named *Ca.* R. shennongii was identified. According to the gene sequence-based criteria for the identification of a new *Rickettsia* species (Fournier et al., 2003), genetic analysis of key genes clearly indicated that these strains represent a novel *Rickettsia* species. Sequences from all strains were almost identical, and they formed distinct clades in the phylogenetic trees. Genetically, this *Rickettsia* is closely related to *R. raoultii*, *R. massiliae*, and *R. conorii* (16S rRNA gene shares 99.91% identity with *R. raoultii* and *R. conorii*, while *groEL* gene shares 99.33% identity with *R. massiliae*), all of which are recognized human pathogens causing spotted fever. Therefore, the pathogenicity of this *Rickettsia* is a concern. Interestingly, this *Rickettsia* was only detected in *R. haemaphysaloides*, a widely distributed three-host tick in China and South Asian countries (Zhou et al., 2006). This species is known to harbor pathogens including *A. phagocytophilum*, *A. ovis*, *R. rhipicephali*, *R. slovaca*, *R. massiliae*, and *Babesia microti* (Kuo et al., 2018; Li et al., 2018; Ghafar et al., 2020; Ali et al., 2021). Our results may suggest the possibility of *R. haemaphysaloides* ticks harboring *Ca.* R. shennongii.

Herein, the DNA of three *Anaplasma* species was detected in all three locations including *Candidatus* Anaplasma boleense, *A. ovis*, and *A. marginale*. *Anaplasma ovis* is a widely-distributed pathogen affecting sheep, goats, and wild ruminants. As the etiological agent of ovine anaplasmosis first reported in 1956, the pathogenicity of *A. ovis* has been well studied since. *Anaplasma ovis* infection in sheep is usually subclinical and mostly manifested as hemolytic anemia (Dahmani et al., 2019). Other main clinical manifestations include extreme weakness, anorexia, and weight loss, but these manifestations mostly occur under poor health conditions (Bauer et al., 2021). In 2010, a human anaplasmosis case caused by an *A. ovis* variant was reported in Cyprus, with clinical symptoms of fever, hepatosplenomegaly, and enlarged lymph nodes (Chochlakis et al., 2010), providing evidence that *A. ovis* might be a potential zoonotic pathogen that may occasionally infect humans. In the present study, ticks from Dehong and Honghe were both tested positive for *A. ovis*, suggesting that *A. ovis* circulation is common in Yunnan Province, and surveillance in local populations is needed.

In this study, *Ca.* A. boleense was detected in ticks from all three sites of Yunnan Province. *Candidatus* Anaplasma boleense was first identified in ticks from Bole City in the Xinjiang Uygur Autonomous Region, Northwest China (Kang et al., 2014). In recent years, it has been detected in mosquitoes and rodents (FJ182047) in multiple provinces of China (Guo et al., 2016). In 2018, *Ca.* A. boleense was found to infect deer, boars, buffalos, cows, and bats in Peninsular Malaysia (Koh et al., 2018). In 2020, *Ca.* A. boleense was reported to infect marsh deer in Argentina, South America (Orozco et al., 2020). Furthermore, some sequences submitted to the GenBank database suggest that this *Anaplasma* exists in Australia (MH500004) and South Africa (MK814450), suggesting that it is distributed worldwide. However, few studies have been performed on the pathogenicity of *Ca.* A. boleense toward humans or other animals. The wide host range, as well as its wide geographical distribution, suggest that this *Anaplasma* species merits further investigation.

*Ehrlichia* is a genus closely related to human diseases. In this study, the DNA of 4 *Ehrlichia* species were identified, including *E. canis*, *E. chaffeensis*, *E. minasensis*, and *Ehrlichia* sp., of which *E. canis* and *E. chaffeensis* are recognized human pathogens (Perez et al., 1996; Bouza-Mora et al., 2017). The most prevalent species was *E. canis* (36.22% positive rate in ticks from Zhaotong), the causative agent of canine monocytic ehrlichiosis. In China, *E. canis* has been detected in various hosts including ticks, goats, dogs, and deer in multiple locations (Xu et al., 2015; Li et al., 2016; Qiu et al., 2016; Yu et al., 2016; Lu et al., 2017; Zhang et al., 2017). Notably, *E. canis* is considered a potential agent of human disease (Perez et al., 1996; Bouza-Mora et al., 2017), although human infection cases have never been reported in China. As shown in Figure 2, the strains we detected are most closely related to *E. canis* strain WHBMXZ-124, a strain identified in *R. microplus* from Wuhan, China. However, considerable genetic distance exists between them, suggesting that they might represent a variant circulating in China. As a recently recognized species closely related to *E. canis*, *E. minasensis* is believed to have evolved from highly variable strains of *E. canis*, and it has been discovered in Canada, Brazil, France, Pakistan, Ethiopia, etc. (Li et al., 2019). Other than ticks, it also infects mammals including cattle and cervid. In China, there is only one report of *E. minasensis* in Hainan Province, South China (Li et al., 2019). In our current study, *E. minasensis* was detected in *R. microplus* ticks from Honghe city, with a high positive rate of 31.07%. To the best of our knowledge, this is only the second report of *E. minasensis* circulating in China. Due to its pathogenicity to mammals, the close relationship to human pathogenic species *E. canis*, and the high infection rate in ticks, its pathogenicity to humans and animals should be further explored.

In summary, this study revealed substantial diversity of *Rickettsia*, *Ehrlichia*, and *Anaplasma* in ticks from Yunnan Province, including a novel *Rickettsia* species. Notably, some of these bacteria are human pathogens (*E. canis*, *E. chaffeensis*, *A. ovis*, etc) and the positive rates are high. Considering the frequent contact between humans and tick hosts (goats and cattle), these results indicate the potential risk of zoonosis transmitted from ticks to humans. Additionally, the human pathogenicity of *Ca.* R. shennongii should be further studied and surveillance in these areas is clearly needed.

## Data availability statement

The datasets presented in this study can be found in online repositories. The names of the repository/repositories and accession number(s) can be found in the article/Supplementary material.

## Ethics statement

This study was approved by the Ethics Committee of the National Institute for Communicable Disease Control and Prevention, Chinese Center for Disease Control and Prevention.

## Author contributions

KL conceived the study. KL and ML designed the experiments. KL, JT, and JH collected the samples. ML and WW performed the experiments. HZ, KL, and WG performed data analysis. HZ, HJ, and KL wrote the manuscript. All authors contributed to the article and approved the submitted version.

## Funding

This work was funded by the National Natural Science Foundation of China (grant no. 82102390), the Key Supporting Scientific Research Projects of Beijing Road Medical Sector, General Hospital of Xinjiang Military Region (2022jzbjl16), the National Key Research and Development Program of China (grant nos. 2020YFA0907101, 2021YFC2301200, and 2021YFC2301202), and the Medical youth top talent project of Hubei.

## Conflict of interest

The authors declare that the research was conducted in the absence of any commercial or financial relationships that could be construed as a potential conflict of interest.

## Publisher’s note

All claims expressed in this article are solely those of the authors and do not necessarily represent those of their affiliated organizations, or those of the publisher, the editors and the reviewers. Any product that may be evaluated in this article, or claim that may be made by its manufacturer, is not guaranteed or endorsed by the publisher.

## Footnotes

^1^ http://www.ncbi.nlm.nih.gov/BLAST
