# Supplementary material for: High diversity of Rickettsia spp., Anaplasma spp., and Ehrlichia spp. in ticks from Yunnan Province, Southwest China
Source: Front Microbiol. 2022 Oct 13;13:1008110. doi: 10.3389/fmicb.2022.1008110 (PMC9606716; doi:10.3389/fmicb.2022.1008110)
Supplement: Supplementary file 1 [file Table_1.DOC]

Table S1. The primers used for amplification of 16S and *gltA* genes from *E. chaffeensis* and *Ca.* A. boleense by nested PCR or semi-nested PCR.

| Primer | Cycle | Bacteria | Gene | Sequence | Anticipated amplicon length |
| --- | --- | --- | --- | --- | --- |
| Echaf-16Sex5 | 1 | *E. chaffeensis* | 16S | 5-CAAGCCTAACACATGCAAGTC-3 | 1200 bp |
| Echaf-16Sex3 | 1 | *E. chaffeensis* | 16S | 5-GTCACTAACCCAACCTTAAATG-3 |
| Echaf-16Sin5 | 2 | *E. chaffeensis* | 16S | 5-ATAATTGTTAGTGGCAGACGG-3 |
| Echaf-16Sin3 | 2 | *E. chaffeensis* | 16S | 5-AGCTTCGAGTTAAGCCAATTC-3 |
| Echaf-gltA-ex5 | 1 | *E. chaffeensis* | gltA | 5-RAATAARGCTGTAGATATTACTTC-3 | 860 bp |
| Echaf-gltA-ex3 | 1 | *E. chaffeensis* | gltA | 5-AGACCAACCAGAYGTTCTWGC-3 |
| Echaf-gltA-in5 | 2 | *E. chaffeensis* | gltA | 5-AATATTAACTTATGATCCAGGATT-3 |
| Echaf-gltA-in3 | 2 | *E. chaffeensis* | gltA | 5-AATCAACATTAGGATACAAAYTAC-3 |
| Abole-gltA-F1 | 1 | *Ca. A. boleense* | gltA | 5-GYAGCATAGCGYATTTGTTGTTG-3 | 670 bp |
| Abole-gltA-F2 | 2 | *Ca. A. boleense* | gltA | 5-TTGAGAGATGAGTATGTYCTACC-3 |
| Abole-gltA-R | 1, 2 | *Ca. A. boleense* | gltA | 5-TCAACRTTAGGGTAAAGCTTGCG-3 |
