# Supplementary material for: High diversity of Rickettsia spp., Anaplasma spp., and Ehrlichia spp. in ticks from Yunnan Province, Southwest China
Source: Front Microbiol. 2022 Oct 13;13:1008110. doi: 10.3389/fmicb.2022.1008110 (PMC9606716; doi:10.3389/fmicb.2022.1008110)
Supplement: Supplementary file 2 [file Table_2.DOCX]

Table S2 Genbank numbers of *Rickettsia*, *Anaplasma* and *Ehrlichia* sequences obtained in this study.

|  | **Gene** | **Genbank numbers** | **Bacterial strain** |
| --- | --- | --- | --- |
| 1 | 16S | OL855727 | Cadidatus Rickettsia shennongii-Honghe-3 |
| 2 | 16S | OL855728 | Cadidatus Rickettsia shennongii-Honghe-94 |
| 3 | 16S | OL855729 | Cadidatus Rickettsia shennongii-Honghe-71 |
| 4 | 16S | OL855730 | Cadidatus Rickettsia shennongii-Dehong-13 |
| 5 | 16S | OL855731 | Cadidatus Rickettsia shennongii-Dehong-17 |
| 6 | 16S | OL855732 | Cadidatus Rickettsia jingxinensis-Dehong-63 |
| 7 | 16S | OL855733 | Cadidatus Rickettsia jingxinensis-Dehong-12 |
| 8 | 16S | OL855734 | Cadidatus Rickettsia jingxinensis-Dehong-26 |
| 9 | 16S | OL855735 | Cadidatus Rickettsia jingxinensis-Zhaotong-162 |
| 10 | 16S | OL855736 | Cadidatus Rickettsia jingxinensis-Zhaotong-168 |
| 11 | 16S | OL855737 | Cadidatus Rickettsia jingxinensis-Honghe-10 |
| 12 | 16S | OL855738 | Cadidatus Rickettsia jingxinensis-Honghe-33 |
| 13 | 16S | OL855739 | Cadidatus Rickettsia jingxinensis-Honghe-72 |
| 14 | ompA | OL856102 | Cadidatus Rickettsia shennongii-Honghe-3 |
| 15 | ompA | OL856104 | Cadidatus Rickettsia shennongii-Honghe-94 |
| 16 | ompA | OL856103 | Cadidatus Rickettsia shennongii-Honghe-71 |
| 17 | ompA | OL856100 | Cadidatus Rickettsia shennongii-Dehong-13 |
| 18 | ompA | OL856101 | Cadidatus Rickettsia shennongii-Dehong-17 |
| 19 | ompA | OL856110 | Cadidatus Rickettsia jingxinensis-Dehong-63 |
| 20 | ompA | OL856118 | Cadidatus Rickettsia jingxinensis-Dehong-12 |
| 21 | ompA | OL856119 | Cadidatus Rickettsia jingxinensis-Dehong-26 |
| 22 | ompA | OL856111 | Cadidatus Rickettsia jingxinensis-Zhaotong-162 |
| 23 | ompA | OL856112 | Cadidatus Rickettsia jingxinensis-Zhaotong-168 |
| 24 | ompA | OL856106 | Cadidatus Rickettsia jingxinensis-Honghe-10 |
| 25 | ompA | OL856105 | Cadidatus Rickettsia jingxinensis-Honghe-33 |
| 26 | ompA | OL856107 | Cadidatus Rickettsia jingxinensis-Honghe-72 |
| 27 | gltA | OL856115 | Cadidatus Rickettsia shennongii-Honghe-3 |
| 28 | gltA | OL856117 | Cadidatus Rickettsia shennongii-Honghe-94 |
| 29 | gltA | OL856116 | Cadidatus Rickettsia shennongii-Honghe-71 |
| 30 | gltA | OL856113 | Cadidatus Rickettsia shennongii-Dehong-13 |
| 31 | gltA | OL856114 | Cadidatus Rickettsia shennongii-Dehong-17 |
| 32 | gltA | OL856120 | Cadidatus Rickettsia jingxinensis-Dehong-63 |
| 33 | gltA | OL856118 | Cadidatus Rickettsia jingxinensis-Dehong-12 |
| 34 | gltA | OL856119 | Cadidatus Rickettsia jingxinensis-Dehong-26 |
| 35 | gltA | OL856121 | Cadidatus Rickettsia jingxinensis-Zhaotong-162 |
| 36 | gltA | OL856122 | Cadidatus Rickettsia jingxinensis-Zhaotong-168 |
| 37 | gltA | OL856123 | Cadidatus Rickettsia jingxinensis-Honghe-10 |
| 38 | gltA | OL856124 | Cadidatus Rickettsia jingxinensis-Honghe-33 |
| 39 | gltA | OL856125 | Cadidatus Rickettsia jingxinensis-Honghe-72 |
| 40 | groEL | OL856128 | Cadidatus Rickettsia shennongii-Honghe-3 |
| 41 | groEL | OL856130 | Cadidatus Rickettsia shennongii-Honghe-94 |
| 42 | groEL | OL856129 | Cadidatus Rickettsia shennongii-Honghe-71 |
| 43 | groEL | OL856126 | Cadidatus Rickettsia shennongii-Dehong-13 |
| 44 | groEL | OL856127 | Cadidatus Rickettsia shennongii-Dehong-17 |
| 45 | groEL | OL856131 | Cadidatus Rickettsia jingxinensis-Dehong-63 |
| 46 | groEL | OL856133 | Cadidatus Rickettsia jingxinensis-Dehong-12 |
| 47 | groEL | OL856132 | Cadidatus Rickettsia jingxinensis-Dehong-26 |
| 48 | groEL | OL856137 | Cadidatus Rickettsia jingxinensis-Zhaotong-162 |
| 49 | groEL | OL856138 | Cadidatus Rickettsia jingxinensis-Zhaotong-168 |
| 50 | groEL | OL856135 | Cadidatus Rickettsia jingxinensis-Honghe-10 |
| 51 | groEL | OL856134 | Cadidatus Rickettsia jingxinensis-Honghe-33 |
| 52 | groEL | OL856136 | Cadidatus Rickettsia jingxinensis-Honghe-72 |
| 53 | 16S | OL855693 | Anaplasma ovis-Honghe-5 |
| 54 | 16S | OL855694 | Anaplasma ovis-Dehong-16 |
| 55 | 16S | OL855695 | Anaplasma ovis-Dehong-81 |
| 56 | 16S | OL855696 | Anaplasma marginale-Zhaotong-47 |
| 57 | 16S | OL855697 | Anaplasma marginale-Dehong-5 |
| 58 | 16S | OL855698 | Anaplasma marginale-Dehong-8 |
| 59 | 16S | OL855699 | Anaplasma marginale-Honghe-56 |
| 60 | 16S | OL855700 | Candidatus Anaplasma boleense-Zhaotong-13 |
| 61 | 16S | OL855701 | Candidatus Anaplasma boleense-Honghe-60 |
| 62 | 16S | OL855702 | Candidatus Anaplasma boleense-Honghe-74 |
| 63 | gltA | OL907262 | Anaplasma ovis-Honghe-5 |
| 64 | gltA | OL907260 | Anaplasma ovis-Dehong-16 |
| 65 | gltA | OL907261 | Anaplasma ovis-Dehong-81 |
| 66 | gltA | OL907265 | Anaplasma marginale-Zhaotong-47 |
| 67 | gltA | OL907263 | Anaplasma marginale-Dehong-5 |
| 68 | gltA | OL907264 | Anaplasma marginale-Dehong-8 |
| 69 | gltA | OL907266 | Anaplasma marginale-Honghe-56 |
| 70 | gltA | OL907267 | Candidatus Anaplasma boleense-Honghe-60 |
| 71 | gltA | OL907268 | Candidatus Anaplasma boleense-Honghe-74 |
| 72 | groEL | OL907276 | Anaplasma ovis-Honghe-5 |
| 73 | groEL | OL907277 | Anaplasma ovis-Dehong-16 |
| 74 | groEL | OL907278 | Anaplasma ovis-Dehong-81 |
| 75 | groEL | OL907272 | Anaplasma marginale-Zhaotong-47 |
| 76 | groEL | OL907269 | Anaplasma marginale-Dehong-5 |
| 77 | groEL | OL907270 | Anaplasma marginale-Dehong-8 |
| 78 | groEL | OL907271 | Anaplasma marginale-Honghe-56 |
| 79 | groEL | OL907273 | Candidatus Anaplasma boleense-Zhaotong-13 |
| 80 | groEL | OL907275 | Candidatus Anaplasma boleense-Honghe-60 |
| 81 | groEL | OL907274 | Candidatus Anaplasma boleense-Honghe-74 |
| 82 | 16S | OL838191 | Ehrlichia minasensis-Honghe-25 |
| 83 | 16S | OL838192 | Ehrlichia minasensis-Honghe-42 |
| 84 | 16S | OL838193 | Ehrlichia sp.-Honghe-12 |
| 85 | 16S | OL838194 | Ehrlichia sp.-Dehong-86 |
| 86 | 16S | OL838195 | Ehrlichia sp.-Honghe-96 |
| 87 | 16S | OL838196 | Ehrlichia chaffeensis-Zhaotong-45 |
| 88 | 16S | OL838197 | Ehrlichia sp.-Dehong-17 |
| 89 | 16S | OL838198 | Ehrlichia sp.-Dehong-18 |
| 90 | 16S | OL838199 | Ehrlichia canis-Zhaotong-61 |
| 91 | 16S | OL838200 | Ehrlichia canis-Zhaotong-76 |
| 92 | 16S | OL838201 | Ehrlichia canis-Zhaotong-84 |
| 93 | gltA | OL907285 | Ehrlichia minasensis-Honghe-25 |
| 94 | gltA | OL907284 | Ehrlichia minasensis-Honghe-42 |
| 95 | gltA | OL907279 | Ehrlichia sp.-Honghe-12 |
| 96 | gltA | OL907281 | Ehrlichia sp.-Dehong-86 |
| 97 | gltA | OL907282 | Ehrlichia sp.-Honghe-96 |
| 98 | gltA | OL907289 | Ehrlichia chaffeensis-Zhaotong-45 |
| 99 | gltA | OL907283 | Ehrlichia sp.-Dehong-17 |
| 100 | gltA | OL907280 | Ehrlichia sp.-Dehong-18 |
| 101 | gltA | OL907287 | Ehrlichia canis-Zhaotong-61 |
| 102 | gltA | OL907288 | Ehrlichia canis-Zhaotong-76 |
| 103 | gltA | OL907286 | Ehrlichia canis-Zhaotong-84 |
| 104 | groEL | OL907296 | Ehrlichia minasensis-Honghe-25 |
| 105 | groEL | OL907297 | Ehrlichia minasensis-Honghe-42 |
| 106 | groEL | OL907295 | Ehrlichia sp.-Honghe-12 |
| 107 | groEL | OL907291 | Ehrlichia sp.-Dehong-86 |
| 108 | groEL | OL907299 | Ehrlichia sp.-Honghe-96 |
| 109 | groEL | OL907292 | Ehrlichia chaffeensis-Zhaotong-45 |
| 110 | groEL | OL907298 | Ehrlichia sp.-Dehong-17 |
| 111 | groEL | OL907290 | Ehrlichia sp.-Dehong-18 |
| 112 | groEL | OL907293 | Ehrlichia canis-Zhaotong-61 |
| 113 | groEL | OL907300 | Ehrlichia canis-Zhaotong-76 |
| 114 | groEL | OL907294 | Ehrlichia canis-Zhaotong-84 |
| 115 | ompB | ON015826 | Cadidatus Rickettsia shennongii-Honghe-3 |
| 116 | ompB | ON015827 | Cadidatus Rickettsia shennongii-Honghe-71 |
| 117 | ompB | ON015828 | Cadidatus Rickettsia shennongii-Dehong-13 |
| 118 | ompB | ON015829 | Cadidatus Rickettsia shennongii-Dehong-17 |
| 119 | sca4 | ON015832 | Cadidatus Rickettsia shennongii-Honghe-3 |
| 120 | sca4 | ON015833 | Cadidatus Rickettsia shennongii-Honghe-71 |
| 121 | sca4 | ON015834 | Cadidatus Rickettsia shennongii-Honghe-94 |
| 122 | sca4 | ON015830 | Cadidatus Rickettsia shennongii-Dehong-13 |
| 123 | sca4 | ON015831 | Cadidatus Rickettsia shennongii-Dehong-17 |
