# Supplementary material for: High diversity of Rickettsia spp., Anaplasma spp., and Ehrlichia spp. in ticks from Yunnan Province, Southwest China
Source: Front Microbiol. 2022 Oct 13;13:1008110. doi: 10.3389/fmicb.2022.1008110 (PMC9606716; doi:10.3389/fmicb.2022.1008110)
Supplement: Supplementary file 3 [file Table_3.DOCX]

Table S1. Tick species, quantity, and vertebrate hosts in different sampling sites, Yunnan Province.

| Location | Host | Tick species and quantity |
| --- | --- | --- |
| Ruili City, Dehong Prefecture | goat | 30 *Rhipicephalus haemaphysaloides* |
|  | cattle | 1 *Rhipicephalus haemaphysaloides* |
|  | cattle | 97 *Rhipicephalus microplus* |
| Zhaoyang District, Zhaotong City | goat | 136 *Rhipicephalus microplus* |
|  | cattle | 60 *Rhipicephalus microplus* |
| Shiping County, Honghe City | goat | 5 *Rhipicephalus haemaphysaloides* |
|  | goat | 15 *Rhipicephalus microplus* |
|  | cattle | 2 *Rhipicephalus haemaphysaloides* |
|  | cattle | 88 *Rhipicephalus microplus* |
