# Supplementary material for: High diversity of Rickettsia spp., Anaplasma spp., and Ehrlichia spp. in ticks from Yunnan Province, Southwest China
Source: Front Microbiol. 2022 Oct 13;13:1008110. doi: 10.3389/fmicb.2022.1008110 (PMC9606716; doi:10.3389/fmicb.2022.1008110)
Supplement: Supplementary file 4 [file Table_4.DOCX]

|  | Strain Dehong-13 | Strain Dehong-17 | Strain Honghe-3 | Strain Honghe-71 | Strain Honghe-94 |
| --- | --- | --- | --- | --- | --- |
| 16S | 99.91%  *Rickettsia raoultii* isolate Tomsk,  *Rickettsia conorii* str. Malish 7 | 99.91%  *Rickettsia raoultii* isolate Tomsk,  *Rickettsia conorii* str. Malish 7 | 99.91%  *Rickettsia raoultii* isolate Tomsk,  *Rickettsia conorii* str. Malish 7 | 99.91%  *Rickettsia raoultii* isolate Tomsk,  *Rickettsia conorii* str. Malish 7 | 99.91%  *Rickettsia raoultii* isolate Tomsk,  *Rickettsia conorii* str. Malish 7 |
| *gltA* | 99.40%  *Rickettsia massiliae* MTU5  *Rickettsia rhipicephali* str. HJ#5 | 99.40%  *Rickettsia massiliae* MTU5  *Rickettsia rhipicephali* str. HJ#5 | 99.30%  *Rickettsia massiliae* MTU5  *Rickettsia rhipicephali* str. HJ#5 | 99.40%  *Rickettsia massiliae* MTU5  *Rickettsia rhipicephali* str. HJ#5 | 99.40%  *Rickettsia massiliae* MTU5  *Rickettsia rhipicephali* str. HJ#5 |
| *groEL* | 99.42%  *R. rhipicephali* str. HJ#5 | 99.42%  *R. rhipicephali* str. HJ#5 | 99.42%  *R. rhipicephali* str. HJ#5 | 99.42%  *R. rhipicephali* str. HJ#5 | 99.42%  *R. rhipicephali* str. HJ#5 |
| *ompA* | 98.31%  *Rickettsia rhipicephali* str. 3-7-female6-CWPP | 98.31%  *Rickettsia rhipicephali* str. 3-7-female6-CWPP | 98.31%  *Rickettsia rhipicephali* str. 3-7-female6-CWPP | 98.31%  *Rickettsia rhipicephali* str. 3-7-female6-CWPP | 98.31%  *Rickettsia rhipicephali* str. 3-7-female6-CWPP |
| *ompB* | 98.16%  *Rickettsia rhipicephali* str. 3-7-6 | 98.14%  *Rickettsia rhipicephali* str. 3-7-6 | 98.20%  *Rickettsia rhipicephali* str. 3-7-6 | 98.22%  *Rickettsia rhipicephali* str. 3-7-6 | — |
| *sca4* | 98.29%  *Rickettsia rhipicephali* str. 3-7-female6-CWPP | 98.37%  *Rickettsia rhipicephali* str. 3-7-female6-CWPP | 98.14%  *Rickettsia rhipicephali* str. 3-7-female6-CWPP | 98.10%  *Rickettsia rhipicephali* str. 3-7-female6-CWPP | 98.14%  *Rickettsia rhipicephali* str. 3-7-female6-CWPP |

Table S3. Nucleotide identity of 16S, *gltA*, *groEL*, *ompA*, *ompB*, and *sca4* genes of the “*Candidatus* Rickettsia shennongii” strains to formally validated *Rickettsia* strains in the Genbank Database.
