# Supplementary material for: High diversity of Rickettsia spp., Anaplasma spp., and Ehrlichia spp. in ticks from Yunnan Province, Southwest China
Source: Front Microbiol. 2022 Oct 13;13:1008110. doi: 10.3389/fmicb.2022.1008110 (PMC9606716; doi:10.3389/fmicb.2022.1008110)
Supplement: Supplementary file 5 [file Table_5.DOCX]

Table S4. Prevalence of Rickettsiales bacteria in male, female, and nymph ticks from Zhaotong city of Yunnan Province, Southwest China.

|  | *R. microplus* | | |  |
| --- | --- | --- | --- | --- |
|  | Male | Female | Nymph | Total |
| *Ca.* Rickettsia jingxinensis | 56/59 (94.92) | 106/133 (79.70%) | 4/4 (100%) | 166/196 (84.69%) |
| *Ca.* Rickettsia shennongii | 0/59 (0.00%) | 0/133 (0.00%) | 0/4 (0.00%) | 0/196 (0.00%) |
| *Ca.* Anaplasma boleense | 0/59 (0.00%) | 1/133 (0.75%) | 0/4 (0.00%) | 1/196 (0.51%) |
| *Anaplasma ovis* | 0/59 (0.00%) | 0/133 (0.00%) | 0/4 (0.00%) | 0/196 (0.00%) |
| *Anaplasma marginale* | 2/59 (3.39%) | 5/133 (3.76%) | 1/4 (25.00%) | 8/196 (4.08%) |
| *Ehrlichia canis* | 0/59 (0.00%) | 71/133 (53.38%) | 0/4 (0.00%) | 71/196 (36.22%) |
| *Ehrlichia* *chaffeensis* | 0/59 (0.00%) | 1/133 (0.75%) | 0/4 (0.00%) | 1/196 (0.51%) |
| *Ehrlichia* *minasensis* | 0/59 (0.00%) | 0/133 (0.00%) | 0/4 (0.00%) | 0/196 (0.00%) |
| *Ehrlichia* sp. | 0/59 (0.00%) | 0/133 (0.00%) | 0/4 (0.00%) | 0/196 (0.00%) |
